# Artificial Rainfall on Grain Quality and Baking Characteristics of Winter Wheat Cultivars in Korea

**DOI:** 10.3390/foods13111679

**Published:** 2024-05-27

**Authors:** Hyeonjin Park, Jin-Kyung Cha, So-Myeong Lee, Youngho Kwon, Jisu Choi, Jong-Hee Lee

**Affiliations:** Department of Southern Area Crop Science, National Institute of Crop Science, Rural Development Administration (RDA), Miryang 50424, Republic of Korea; tinapark@korea.kr (H.P.); jknzz5@korea.kr (J.-K.C.); olivetti90@korea.kr (S.-M.L.); kwon6344@korea.kr (Y.K.); choijisu24@korea.kr (J.C.)

**Keywords:** wheat, artificial rainfall, pre-harvest sprouting, flour quality

## Abstract

Wheat (*Triticum aestivum* L.) stands as a significant cereal crop globally, including in Korea, where its consumption reached 35.7 kg per capita in 2023. In the southern regions of Korea, wheat cultivation follows paddy rice, with harvesting typically occurring during the rainy season in mid-June. This timing, coupled with the high humidity and unpredictable rainfall, often leads to pre-harvest sprouting and subsequent deterioration in flour quality. To assess the impact of rain on flour quality, an artificial rain treatment was administered 45 days after heading in an open field greenhouse, followed by flour quality analysis. The color measurement revealed an increase in the L* parameter, indicative of enhanced kernel vitreousness, attributed to endosperm starch degradation via alpha-amylase activation induced by water absorption. Moreover, significant changes were observed in ash content and the gluten index within the wetted group, resulting in decreased dough strength and stability, ultimately leading to a reduction in loaf volume. Consequently, it is recommended that wheat be harvested 4–7 days after reaching the physiological maturity stage to avoid the rainy season and ensure the production of high-quality wheat.

## 1. Introduction

Cereals are a significant primary food source worldwide, playing a crucial role in the diets of millions of people across various cultures and regions. Wheat (*Triticum aestivum*) holds the second position in importance after rice and stands as the most extensively grown cereal worldwide, serving as a cornerstone of food security [1,2].

Despite its crucial role, wheat is susceptible to significant yield setbacks from abiotic stresses, with yield penalties reaching as high as 82.1% [3]. Annual world wheat yields vary widely, from less than 1 t/ha in conditions where water or nutrients are limited to over 10 t/ha in cooler, well-watered growing environments [2]. For instance, Australia, one of the largest wheat producers, exhibited a 5-year average yield of 1.92 t/ha from 2016 to 2020. However, the average yield ranged from 2.61 t/ha in 2017 to 1.47 t/ha in 2020, demonstrating the significant impact of weather conditions on wheat yields [4,5]. Environmental conditions exert a greater influence on wheat quality than on genotype quality [6,7,8]. The influence of environmental factors on wheat quality exceeds that of genetic variation by a factor of 5 to 6 times [9].

In Korea, the winter wheat–rice rotation constitutes the predominant double-cropping system, leading to overlaps between the wheat grain-filling period and the rainy season. Rainfall exposure during grain-filling can lead to germination and quality deterioration, triggering pre-harvest sprouting (PHS) [10]. PHS is directly associated with reductions in both wheat quality and yield and is prevalent in areas experiencing rainfall during the harvesting period.

PHS can lead to significant financial losses, as the value of sprouted wheat loses declines by 20–50%. This grain is often unsuitable for human consumption, diverted instead to animal feed [11,12]. Global annual losses in both the yield and quality of wheat attributable to PHS approximate $1 billion. Flour produced from sprouted wheat often exhibits diminished brightness attributed to increased ash content, leading to an off-color appearance in noodles and bread [13]. Sprout damage is initiated by the enzyme alpha-amylase, which breaks down long starch chains in the wheat endosperm into shorter fragments, ultimately leading to inferior quality in bread, cake, and noodles [11,14].

Resistance to PHS is associated with various morphological features of the spike, including the angle of awn, waxiness, and hairiness, as well as genetic factors like seed dormancy (*MFT1*) and the seed coat color gene (*Myb10*) [15,16,17]. However, the influence of environmental factors on pre-harvest sprouting surpasses that of genetic variation. Thus, even PHS-resistant cultivars can germinate under favorable weather conditions, with PHS more significantly triggered under cooler conditions coupled with rainfall compared to warmer conditions [18]. PHS typically happens following the maturation of the wheat kernel; however, germination may occur as early as 18 days after anthesis when exposed to favorable conditions [19].

During germination, the aleurone layer synthesizes and secretes alpha-amylases, which break down starch and cell wall materials in the endosperm. This process, crucial for crop growth, can lead to PHS when it occurs on the mother plant before harvest due to weather conditions [20,21].

The Hagberg–Perten falling number (FN) method identifies starch degradation caused by alpha-amylase enzyme activity in flour [22]. Introduced in the 1960s, this test assesses alpha-amylase activity resulting from sprout damage in wheat. In the United States, a typical cutoff value for the FN is 300 s. Grains with an FN below 300 s receive a discount of $10 per metric ton for every deduction of 25 s. Moreover, the detection of low FN in Australia leads to a monetary loss ranging from $20 to $50 per ton [23,24]. Wheat grades in Korea are based on protein and ash content, as well as liter weight, crucial for determining bread quality.

Seed storage proteins play a crucial role in determining the end-use quality and manufacturing processes of various wheat-based products [25,26]. Despite the significant influence of PHS on proteins, wheat protein content alone cannot fully explain grain defects caused by rain. Instead, starch degradation, ash content, and SDS sedimentation are the most affected by direct moisture exposure on the spikes [27]. Wheat reaches physiological maturity 45 days after heading, during which period it is vulnerable to quality deterioration due to rain. Therefore, this period is optimal for rain treatment in wheat quality research [27].

While there have been numerous studies on PHS, there is limited research on grain quality deterioration caused by rain before germination at maturity. Previous studies have shown no significant correlation between wheat protein and rain treatment [27]. This raises questions regarding whether grain quality parameters such as protein content can explain quality deterioration. This study aims to investigate the specific impacts of artificial rainfall on the grain and flour quality of wheat during the critical maturation phase. By focusing on controlled artificial rainfall, we seek to simulate adverse weather conditions and understand the extent of quality deterioration in Korean wheat cultivars. This research provides empirical evidence that can inform agricultural practices and breeding programs to mitigate pre-harvest sprouting (PHS) and related quality losses.

## 2. Materials and Methods

### 2.1. Plant Materials

Two winter wheat cultivars, Jokyoung and Hwanggeumal, were selected for their contrasting seed coat colors and their prevalence in Korean agriculture. Jokyoung has white grain, which is known to be susceptible to pre-harvest sprouting (PHS), while Hwanggeumal has red grain, which is known to be resistant to PHS. This selection allows for a comparative analysis of the impact of artificial rainfall on different levels of PHS resistance. These two cultivars were sown in the greenhouse located in Miryang, Gyeongsangnam-Do, Korea, on 5 November 2022, with a row spacing of 30 cm. The polyethylene (PE) cover of the greenhouse was removed during the period from sowing until heading to optimize wheat growth by preventing heat stress and lodging.

Before sowing, fertilization was conducted at a ratio of N_2_:P_2_O_5_:K_2_O = 4.5:7.4:3.9 kg/10a as basal dressing and an additional 4.5 kg/10a was applied as top dressing at the jointing stage, following the standard cultivation method for wheat as outlined by the Rural Development Administration [28]. Weeds and pests were controlled as required to prevent yield losses.

### 2.2. Artificial Rain Treatments

To assess the impact of rainfall on the grain quality of wheat, two artificial rain treatments were designed: a control group (no treatment) and a wetted group (rain treatment applied 45 days after heading using a sprinkler system, with a rate of 5 mm/hour). The reasons for choosing this treatment condition are as follows: firstly, the rainy season usually occurs in mid-May, which corresponds to the wheat’s physiological maturity; secondly, 45 days after heading is known as the most vulnerable time for wheat flour quality such as ash content and SDS sedimentation [27]. Therefore, wheat quality becomes more sensitive to direct moisture on spikes during this period. The artificial rainfall lasted for 8 h, and following the treatment, the PE cover was closed to maintain humidity above 90% and prevent additional treatment by natural rainfall.

### 2.3. Flour Sample Preparation

At maturity, spikes from each experimental plot were collected in three replicates and air-dried at room temperature for 3 days, followed by hand-threshing and aspiration to eliminate husks and debris. Subsequently, grains were air-dried in room temperature until grain moisture content reached 14%. Wheat samples were mixed with tempering water, calculated according to the grain moisture content, and tempered at room temperature for 24 h until reaching a final moisture content of 16% [29]. Subsequently, the samples were milled using a Brabender Quadrumat Junior mill (Brabender OHG, Duisburg, Germany) with a 1 mm sieve.

### 2.4. Color Measurements

Grain color was assessed using a spectrophotometer (CM-3500d, Konica Minolta, Tokyo, Japan). The instrument was standardized using a reference plate (L = 97.75, a = 0.49, b = 1.96), and parameters including brightness (L*), redness (a*), and yellowness (b*) were employed. The total color difference (ΔE*ab) was calculated using Equation (1) [30]:

(1) $$∆E=\sqrt{{∆L}^{*2}+{∆a}^{*2}+{∆b}^{*2}}$$

### 2.5. Measurement of Flour Quality

Flour protein content was assessed using the LECO FP628 nitrogen/protein analyzer (Laboratory Equipment Co., St. Joseph, MI, USA), following the AACC method 46-30.01 [31]. Flour ash content was measured using a digital muffle furnace (Daihan Sci. Co., FHX-14, Seoul, Republic of Korea), following the AACC method 08-02.01. Flour dry and wet gluten contents were determined using the Glutomatic 2200 (Perten Instruments AB, Huddinge, Sweden), following the method described in AACC method 38-12.02 [31]. The SDS sedimentation volume was determined according to the AACC method 46-30.01 [31].

### 2.6. Alpha-Amylase Assay and Falling Number

Alpha-Amylase activity was measured using the Amylase Activity Assay Kit (Sigma Aldrich Chemical Co, St. Louis, MO, USA), following the provided instructions. FN is a widely used method for indirectly assessing the starch quality in wheat seeds. The FN values of the control and rain-treated flour were measured using the FN standard AACC method 56-81B [31,32].

### 2.7. Baking Quality

A Mixograph (National Mfg., Lincoln, NE, USA) equipped with a 10-g bowl was utilized to measure dough rheological properties. The mixing speed was set at 88 rpm, and the test duration was 10 min. Water amounts added to each flour sample were determined based on water absorption estimates obtained via near-infrared (NIR) spectroscopy. Mixograph parameters, including MPTi (midline peak time), MPV (midline peak value), MPW (midline peak width), MTxV (midline time x = 8 min value), and MTxW (midline time x = 8 min width), were selected to assess dough quality using MIXSMART software (v.3.8). The flour samples were subjected to a long fermentation straight dough bread-making process, based on AACC-approved methods 10-10.03 [31]. After baking, the loaves were cooled at room temperature for an hour, and loaf volumes were determined using rapeseed displacement [31].

### 2.8. Statistical Analysis

All tests were performed in three replicates. Data were analyzed using SAS 9.2 (SAS Institute Inc., Cary, NC, USA) and Duncan’s multiple range test was performed for the significant difference considering *p*-value < 0.05.

## 3. Results and Discussion

### 3.1. Kernel Quality Characteristics

Vitreous kernels exhibit a glasslike, translucent appearance, while nonvitreous (starchy or chalky) kernels lack translucency and may appear light-colored, facilitating easy differentiation via transverse sectioning. In vitreous endosperm, the adhesion between starch granules and storage proteins is significantly stronger compared to starchy endosperm, resulting in a more densely packed structure. Environmental factors such as temperature, light intensity during grain filling, grain drying rate, drought conditions, and nitrogen fertilization levels influence kernel vitreousness [33,34,35]. Higher vitreousness in wheat grains often correlates with elevated protein content, indicating an impact on processing suitability and quality. Specifically, elevated gliadin content in the endosperm leads to tighter binding between protein matrices and starch during seed drying, reducing porosity and consequently increasing vitreousness [36].

**Figure 1:**
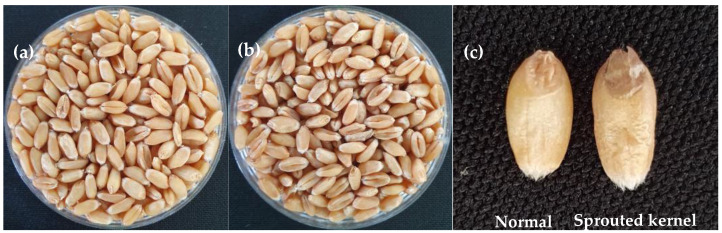
Exterior kernel color changes in the (**a**) control group and (**b**) wetted group. Despite the absence of a significant decrease in grain yield, there was an increased occurrence of *Fusarium* head blight in the wetted group; (**c**) pre-harvest sprouting in the wetted group.

In a previous study, the proportion of vitreous grains decreased from 40 to 55 days after heading, following observed starch degradation in FE-SEM [27]. Color measurement using a colorimeter revealed significant interactions between cultivars and treatments across all parameters. Therefore, treatment effects on the entire cultivars were examined. The results of the color analysis showed that the value of the L* parameter (Figure 1, Table 1), directly related to the lightness of the grain, increased slightly by 2.1% in the wetted plot. The total color difference (ΔE*ab), representing the magnitude of the color difference between the control and wetted plot, was 1.40, indicating that artificial rain caused grain vitreousness in the wetted group (Table 1).

### 3.2. Starch Characteristics

**Figure 2:**
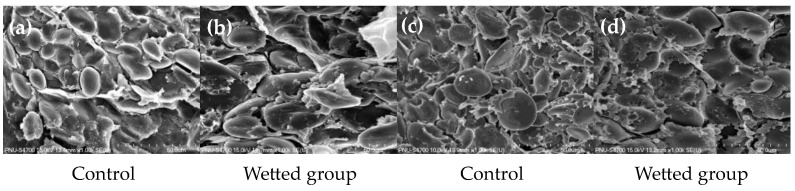
Scanning electron micrographs of transverse sections of (**a**) Jokyoung control, (**b**) Jokyoung wetted, (**c**) Hwanggeumal control, and (**d**) Hwanggeumal wetted. Wheat grains harvested without rain exposure exhibited intact starch granules embedded within a highly dense protein matrix. In the wetted group, however, the starch granules and protein matrix began to break down, leading to the hydrolysis of A- and B-granules and resulting in increased pore formation.

Wheat starch is deposited in the form of water-insoluble semi-crystalline granules in plastids, classified into larger A-type starch granules (≥10 μm) and smaller B-type starch granules (<10 μm) [37]. These develop during different stages of endosperm development. A-granules contribute to higher amylose contents, peak viscosity, and swelling power, while B-granules exhibit higher gelatinization temperatures and lipid contents. These differences in physicochemical properties significantly affect dough characteristics, processing properties, and the quality of end-products. In dough, B-granules fill the interstitial spaces of A-granules, extensively impacting dough rheological properties and bread quality [38,39]. In a previous study, treating wheat plants with artificial rain at maturity, closer to harvest (55 days after heading), led to elevated degradation of starch [27]. Consistent with these findings, B-granules were observed to degrade, and pores were evident on the surface of A-granules, indicating potential deterioration in flour quality (Figure 2).

Continuous rain and high humidity during wheat maturation could lead to pre-harvest sprouting, thereby increasing the synthesis and secretion of hydrolytic enzymes, such as alpha-amylase, which is synthesized in the aleurone layer of wheat and then transported to the endosperm, where it breaks down starch. Even though visible sprouting is not observed in the wetted group, the elevated alpha-amylase levels suggest that grains may initiate germination without apparent signs of sprouting [20,40,41].

The alpha-amylase activity can be indirectly measured using methods such as the Amylograph, Hagberg FN, Rapid Visco Analyser, etc., and in this study, alpha-amylase activity and FN values were measured [42]. Both cultivars ‘Jokyoung’ and ‘Hwanggeumal’ exhibited increased alpha-amylase activity, with values changing from 0.243 to 0.430 and from 0.173 to 0.363, respectively. Also, decreased FN values following rainfall treatment were observed, resulting in an anticipated reduction in dough and baking quality (Table 2).

### 3.3. Flour Quality

The milling rates showed no significant differences depending on the treatment, with an average of 72.0%. To assess the effect of rain on flour quality, protein, ash, amylose, and gluten content, as well as the SDS-sedimentation test, were implemented.

Protein content stands as one of the most crucial factors in determining flour and baking quality, while in Korea, the price of wheat grain is determined based on grain moisture and protein content. It is widely acknowledged that protein content is adversely affected by pre-harvest sprouting [43,44]. However, the findings of this study suggest that even with rainfall treatment, no significant changes in moisture, protein, amylose, and SDS sedimentation could not be observed until after wheat kernel germination (Table 3). It is posited that such criteria could only be applicable after significant progress in germination has occurred.

Direct exposure to moisture during the maturation phase results in the absorption of moisture by the spikes, causing the seed coat to undergo repeated cycles of swelling and drying. Consequently, the seed coat easily detaches from the grain, leading to an increase in ash content [45]. Elevated ash content in flour reduces gluten viscosity, thereby deteriorating processing properties and resulting in darker flour, which adversely affects consumer preference. Ash content exhibited statistically significant results of 0.35% in the control group and 0.43% in the wetted group, suggesting a potential for darker flour and bread (Table 4).

The gluten index (GI) is a measure of wheat protein that provides a simultaneous determination of gluten quality and quantity [46]. Gluten proteins, insoluble storage proteins critical for determining wheat quality, can be divided into two main fractions: gliadins and glutenins. Gliadins, existing as monomers with a molecular weight of 3 to 8 × 10^4^ Da, contribute to the dough’s viscous flow and ductility, while glutenins, forming polymers with a molecular weight ranging from 10^5^ to 10^7^ Da, determine the dough’s viscoelasticity, thus significantly influencing the end-use quality [47,48,49,50]. The gluten index in the wetted group was 82.2, compared to 87.3 in the control group, indicating a deterioration in flour quality and an anticipated decline in dough quality.

### 3.4. Dough and Baking Quality

The mixograph is a rapid tool utilized for assessing the mixing properties of flour, requiring only a small quantity of flour (10 g) and with a short testing duration. Mixograph parameters have demonstrated significant correlation with baking quality, making it useful in wheat-breeding endeavors with an expectation of enhancing baking quality [51,52,53].

To examine the dough characteristics by cultivars and treatment, Mixograph analysis was conducted. The results revealed no statistically significant correlation between cultivars and treatments; therefore, individual testing was performed to assess the influence of each parameter by cultivars and treatments. It was observed that dough development time (MPTi) increased in the wetted group, for both tested cultivars, while dough strength (MPV and MPW) decreased. Dough stability (MTxV), measured by the width of the Mixogram 7 min after the start of mixing, showed no significant difference with rainfall treatment (Table 5). Overall, the experimental findings indicate that rainfall treatment adversely affects dough quality and is anticipated to impact baking suitability.

**Figure 3:**
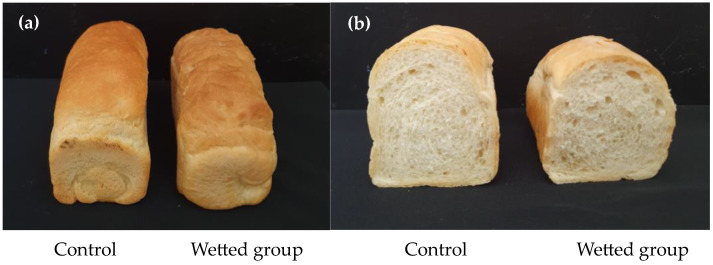
Comparison of (**a**) exterior and (**b**) cross-sections of the loaves produced with control and water-treated ‘Jokyoung’ wheat grains.

To assess the baking quality across cultivars and treatments, bread was prepared following the AACC method 10-10.03 (AACCI, 2010), and measurements were subsequently taken for bread volume and inner crumb color. The findings indicated a reduction in loaf volume within the wetted group, although statistical significance was not observed (Figure 3). Regarding color measurements, it was noted that the inner crumb regions darkened with rainfall treatment, attributed to an increase in ash content within the flour (Table 6).

The period between 40 and 45 days after heading is a critical period that is heavily influenced by rainfall. Exposure to rainfall for more than 8 h during this period is anticipated to result in increased sprouting and moisture content, as well as reduced SDS sedimentation volume, potentially leading to a decline in flour quality [27]. Building on this understanding, an analysis of the effects of rainfall 45 days after heading was conducted.

The period twenty days prior to wheat maturation is a crucial period for breaking dormancy, during which environmental factors such as temperature and humidity play a more significant role than genetic factors in determining dormancy break. According to a previous study [45], up until 10 days prior to maturity, the seed moisture content reaches 51–52%, affecting yield rather than grain quality. However, after this period, a decline in quality begins. The experimental treatments in this study were conducted 45 days after heading (June 2nd for both cultivars), corresponding to 10 days before maturity. In line with prior research, a significant decrease in various aspects of flour quality was observed.

The results of the color measurement indicated an increase in the L* parameter, reflecting kernel vitreousness, as the spike was exposed to rain treatment. This phenomenon may be attributed to endosperm starch degradation due to alpha-amylase activation triggered by moisture absorption. This finding is supported by the increased alpha-amylase assay result and the decreased falling number test result. FE-SEM analysis also revealed the degradation of A- and B-starch granules following artificial rainfall treatment. Moreover, the increased ash content negatively affected flour quality by reducing dough characteristics such as strength and stability.

It is well-established that protein content is adversely affected by pre-harvest sprouting. However, interestingly, no significant decline was observed in this study, possibly because the analysis was conducted at a previous stage before pre-harvest sprouting occurred. It is noteworthy that, 4–7 days after wheat physiological maturity, typically around 45 days after heading, grain moisture reaches 20%, making it suitable for harvesting with a lower risk of quality deterioration. Therefore, timely harvesting and drying are critical to minimize flour quality deterioration and pre-harvest sprouting.

## 4. Conclusions

This study demonstrated that artificial rainfall during the critical maturation phase significantly impacts wheat grain and flour quality. The findings revealed increased alpha-amylase activity, decreased FN values, and deteriorated dough quality in both Jokyoung and Hwanggeumal cultivars. These results underscore the importance of timely harvesting and drying practices to mitigate the adverse effects of pre-harvest sprouting and ensure high-quality wheat production. Further research should focus on developing wheat cultivars with enhanced resistance to abiotic stresses and optimizing agricultural practices to reduce yield losses.

## Figures and Tables

**Table 1:** Wheat kernel color values in the control and wetted groups.

Treatment ^1^	L*	a*	b*	ΔE*ab
Control	48.38 ± 0.23 ^b^	9.42 ± 0.15 ^a^	23.46 ± 0.25 ^b^	-
Wetted	49.41 ± 0.31 ^a^	9.19 ± 0.22 ^b^	23.72 ± 0.18 ^a^	1.40 ± 0.29

Values with a different letter in the same column are significantly different (*p* < 0.05). ^1^ Results of the flour quality analysis showed a significant correlation between cultivar and treatment. Therefore, integrated results of the treatment effect for both cultivars are presented.

**Table 2:** Alpha-amylase assay and falling number test results.

Cultivar	Treatment	Alpha-Amylase Activity (CU(%d.b.))	Falling Number (s)
Jokyoung	Control	0.243 ^b^	415
	Wetted	0.430 ^a^	368
Hwanggeumal	Control	0.173 ^b^	432
	Wetted	0.363 ^a^	424

Values with different letters in the same column and for the same wheat variety are significantly different (*p* < 0.05).

**Table 3:** Effect of artificial rainfall on the protein, amylose, moisture content, and SDS sedimentation value of the tested cultivars.

Cultivar	Treatment	Protein Content (%)	Amylose Content (%)	Moisture (%)	SDS -Sedimentation (mL)
Jokyoung	Control	11.0	16.4	13.1	58.0
	Wetted	11.5	14.3	13.1	56.3
Hwanggeumal	Control	12.2	13.0	13.4	58.5
	Wetted	13.9	13.0	13.2	57.5

No significant differences were observed between the control and wetted groups.

**Table 4:** Effect of artificial rainfall on the flour quality of the tested cultivars.

Treatment ^1^	Ash Content (%)	Gluten Content (g)	Gluten Index
Control	0.345 ^b^	10.3 ^b^	87.3 ^a^
Wetted	0.432 ^a^	11.9 ^a^	82.2 ^b^

Values with different letters in the same column are significantly different (*p* < 0.05). ^1^ Results of the flour quality analysis showed a significant correlation between the cultivar and treatment. Therefore, integrated results of the treatment effects for both cultivars are presented.

**Table 5:** Effect of artificial rainfall on mixographic characteristics of wheat flour.

Cultivar	Treatment	MPTi (min.)	MPV (%)	MPW (%)	MTxV (%)	MTxW (%)
Jokyoung	Control	5.4 ^b^	49.4	15.7 ^a^	40.9 ^b^	8.4
	Wetted	6.7 ^a^	47.0	14.4 ^b^	42.9 ^a^	8.5
Hwanggeumal	Control	4.4 ^b^	49.4	14.5 ^a^	39.5 ^b^	7.3
	Wetted	4.5 ^a^	48.7	13.2 ^b^	40.6 ^a^	7.5

Values with different letters in the same column and for the same wheat variety are significantly different (*p* < 0.05).

**Table 6:** Effect of artificial rainfall on loaf characteristics.

Cultivar	Treatment	Loaf Characteristics		Color Values			
		Mixing Time (min.)	Loaf Volume (mL)	L*	a*	b*	ΔE*ab
Jokyoung	Control	3:25	800 ^a^	80.18 ^a^	0.41 ^b^	18.56 ^a^	-
	Wetted	3:06	670 ^b^	79.37 ^b^	0.76 ^a^	17.50 ^b^	1.38
Hwanggeumal	Control	2:53	600 ^a^	78.77 ^a^	0.63 ^b^	17.55 ^b^	-
	Wetted	2:51	580 ^b^	76.77 ^b^	0.87 ^a^	18.13 ^a^	2.10

Values with different letters in the same column and for the same wheat variety are significantly different (*p* < 0.05).

## Data Availability

The original contributions presented in the study are included in the article, further inquiries can be directed to the corresponding author.
